# A comparison of the effects of remote coaching HIIT training and combined exercise training on the physical and mental health of university students

**DOI:** 10.3389/fpsyg.2023.1182332

**Published:** 2023-05-12

**Authors:** Yu Wang, Ningxin Jia, Yanan Zhou, Linlin Fu, Lixia Fan, Bin Li

**Affiliations:** College of Physical Education, Shandong Normal University, Jinan, China

**Keywords:** HIIT, combined exercise training, mental health, university students, remote coaching

## Abstract

**Aim:**

To compare the differences in the effects of based on remote coached high intensity interval training and combined exercise training on the physical and mental health of university students.

**Method:**

Sixty university students were recruited from Shandong Normal University and randomly divided into HIIT group (*n* = 30) and AR group (*n* = 30), with the HIIT group using high-intensity interval training intervention and the AR group using combined exercise (aerobic combined with resistance) training intervention for 8 weeks. Mental health indicators, fitness indicators and body composition indicators were measured at the beginning and end of the intervention.

**Results:**

After 8 weeks, among the mental health indicators, the results of the Symptom Self-Rating Scale (SCL-90) test showed a significant improvement in the HIIT group in terms of total score, somatization, obsessive-compulsive, interpersonal sensitivity, depression, hostility, and psychoticism (*p* < 0.05); the AR group showed significant improvements in psychoticism (*P* < 0.05). There were no significant differences between the two groups. The results of the Pittsburgh Sleep Quality Index scale (PSQI) showed a significant difference in sleep efficiency in the HIIT group with an inverse improvement in scores; the AR group showed no significant improvement in each test item. The results of the between-group covariance showed significant differences in sleep efficiency and hypnotic drugs in the HIIT group (p < 0.05). Among the fitness indicators, the HIIT group showed significant improvements in maximum oxygen uptake, grip strength and flexibility (*P* < 0.05); the AR group showed significant improvements in back muscle strength and flexibility (*P* < 0.05). The results of the between-group covariance showed significant improvements in maximum oxygen uptake in the HIIT group (*P* < 0.01). Regarding body composition indicators, there was a significant improvement in Body weight, BMI, Body fat percentage and Waist-to-hip ratio in both the HIIT and AR groups (*P* < 0.01). There were no significant differences between the two groups.

**Conclusion:**

HIIT and combined exercise training based on remote coaching had some improvement on fitness level and body composition of university students, HIIT was more advantageous in improving aerobic endurance, and HIIT based on remote coaching may have better effect than combined exercise in mental health.

**Trial registration:**

Chinese Clinical Trial Register, ChiECRCT20220149. Registered on 16 May 2022.

## 1. Introduction

In recent years, the effective enhancement of the physical fitness and mental health of university students has become a hot issue of concern in the field of education and sports. University students are in a period of challenge, risk and social development transition (Feng et al., 2014). This period can have a significant impact on adapting and maintaining healthy behaviors (VanKim and Nelson, 2013). Physical health is one of the important indicators affecting health. It is found that the physical health level of college students in China continues to decline (Dong et al., 2023). Physical health problems of college students have become a common problem in our universities. The “Mental Quality Training for College Students” group conducted a mental health examination on nearly 6,000 college students from 23 universities in China. The results show that about 16.51% of Chinese university graduates have serious mental health problems (Zhu and Li, 2022). Approximately 10–20% of adolescents worldwide suffer from mental health problems, and the number of university students with psychological problems continues to rise worldwide (Hassanzadeh et al., 2019). The pressure of changing living environments and lifestyles, intense study and work, heavy financial burdens and complex employment situations have led to a high incidence of mental health problems among university students (Cuijpers et al., 2016; Al Bahhawi et al., 2018; Fawaz and Samaha, 2021). In addition, with the advent of the Internet era, various online games, online social platforms and online information have brought more serious challenges to the mental health of university students (Shen et al., 2020). Undoubtedly, poor mental health not only makes academic adjustment difficult for university students, but also affects their academic commitment and performance, leading to negative consequences such as repeating a grade or dropping out of school (Mohammad et al., 2020; Wasil et al., 2022). It can also undermine quality of life and wellbeing (Ridner et al., 2016) and even hinder social adjustment and career development (Lei et al., 2016).

Research shows that physical activity among university students has decreased significantly (VanKim and Nelson, 2013), and physical inactivity is the greatest public health problem of the 21st century (Ghrouz et al., 2019). Therefore, ensuring daily participation in physical activity is an important public health challenge for university students (Dong, 2017). Physical activity has been well documented to promote the physical health of university students (Kim et al., 2022; Tsai and Hsu, 2022). High-intensity interval training can effectively improve college students' cardiorespiratory fitness and endurance of physical activity, and can effectively improve their cognitive function (Cooper et al., 2018). There is also strong evidence that regular physical activity can improve a range of mental health problems (e.g., self-esteem, anxiety and depression) (Jia et al., 2021; Yan et al., 2022). Since the early 1980s, when Kobasa et al. (1982), an American psychologist, proposed the idea that physical exercise affects psychological stress, researchers have gradually begun to focus on the effects and comparisons of the time, frequency and intensity of physical exercise and the interaction between the three on the psychological stress of university students, while the results of program differences in exercise interventions for university students' psychological stress are relatively rare.

In 2020, the new coronavirus swept the world, due to the rapid spread of the new coronavirus, infectious and other characteristics, people's traditional exercise methods are affected to a certain extent, the advantages of remote coaching exercise highlights, remote coaching exercise is not affected by time and space, can strengthen the body and improve the quality of life of people (Buneviciene et al., 2022; Dadswell et al., 2022; Han et al., 2022). Previous studies have shown that online combined exercise interventions are better for improving mental health and quality of life in obese children (Ding et al., 2021). There are also studies that confirm that tele-exercise interventions improve muscle strength, cardiorespiratory endurance and mental health in breast cancer patients (Dong et al., 2018, 2019, 2020). HIIT is currently an emerging form of physical exercise that is more popular among college student populations, while joint training is a more traditional form of exercise that is chosen by more people. However, less research has been conducted on the fitness effects of both exercise modalities based on remote coaching. In this study, a randomized parallel controlled study was conducted to compare the advantages and disadvantages of remote coaching HIIT and combined training on physical and mental health promotion among university students.

## 2. Methodology

### 2.1. Object of study

The participants for this study were recruited from Shandong Normal University, and 60 participants were planned to be recruited.

Inclusion criteria: Healthy male and female university students aged ≥ 18 years; BMI of 18.5–23.9 non-athletic majors.

Exclusion criteria: (1) major cardiovascular, metabolic or endocrine diseases; (2) smokers; (3) heavy drinkers (>7 drinks per week for women and >14 drinks per week for men); (4) pregnant or planning to become pregnant; (5) diagnosed psychological disorders such as depressive disorders and anxiety disorders.

The HIIT group performed exercises according to high-intensity interval training, and the combined exercise group (AR group) performed exercises according to aerobic combined with resistance. The interventionists in this trial were two physical education practitioners with formal normal education who did not take the test for the outcomes of interest. The results of interest for the HIIT and AR groups were assessed by the same testers who underwent relevant research and training in advance to ensure the accuracy of the tests.

### 2.2. Interventions

Participants in the HIIT group performed 40 min high-intensity interval training three times a week, with 5 min of preparation, 30 min of HIIT and 5 min of relaxation, for a total of 8 weeks of exercise intervention. The training was concentrated throughout the day, and the participants could choose their own suitable exercise time, participating in a maximum of one exercise session per day and ensuring three exercise sessions per week. Throughout the exercise sessions, each participant is given three opportunities to take leave, and absences beyond three are treated as shedding. The interventionist mapped out the schedule of the exercise intervention sessions for the following day in the WeChat group 1 day in advance and distributed an online form with videos of specific aerobic resistance exercises to the WeChat group for the participants' initial understanding and learning. Participants can choose to participate in the intervention according to their own schedules, and each participant's weekly participation is recorded according to the daily tally sheet, which records attendance. Participants will wear an exercise bracelet during training to monitor exercise intensity. Exercise intensity is increased every fortnight, mainly by increasing the difficulty of the movements and by reducing the amount of rest time in between. During the 8 weeks, the participants are given remote video intervention via a remote session app using a mobile phone or computer to supervise them throughout the training process and to give them timely and effective encouragement to complete this training if they are slacking off.

Participants in the AR group performed 40 min aerobic resistance training three times a week with 5 min of preparation, 15 min of aerobic exercise, 15 min of elastic band resistance exercise and 5 min of relaxation for a total of 8 weeks of exercise intervention. Other exercise instruction and supervision measures were the same as in the HIIT group.

### 2.3. Indicators

The results were tested separately before and after the 8-week intervention. The students were tested by professionals from Shandong Normal University in the form of a questionnaire, which the students completed according to their actual situation.

The main indicators studied in this study include Mental health indicators [Symptom Self-Rating Scale (SCL-90), Pittsburgh Sleep Quality Index scale (PSQI)]. Physical fitness indicators (Maximum oxygen uptake, Muscle strength, Flexibility) and Body composition indicators (Body weight, BMI, Body fat Percentage, Lean weight, Body hydration, Waist-to-hip ratio).

#### 2.3.1. Mental health indicators

Using the Symptom Self-Rating Scale (SCL-90) (Derogatis et al., 1973). To assess the individual mental health status of university students before and after the intervention, the scale consists of 90 items and contains a relatively wide range of psychiatric symptomatology in 90 items, covering a wide range of aspects such as somatization, obsessive-compulsive, interpersonal sensitivity, depression, anxiety, hostility, phobic anxiety, paranoiel ideation, psychoticism, and other (sleep disorders and poor eating).

The Pittsburgh Sleep Quality Index (PSQI) was used (Buysse et al., 1989). The PSQI is used to assess the subjective sleep quality participants in the last month. Seven tests were used to assess subjective sleep quality, sleeping time, subjective sleep duration, sleep efficiency, sleep disorders, hypnotic drugs and daytime dysfunction.

#### 2.3.2. Physical fitness indicators

Maximum oxygen uptake was measured in all participants before and after the intervention using the Bruce running table test protocol (modified Bruce protocol). Participants' grip strength was measured using a grip strength device and participants' back muscle strength was measured using an electronic back muscle strength tester. The flexibility of the participants was measured using seated forwards bending.

#### 2.3.3. Body composition indicators

Body weight, BMI, Body fat percentage, lean weight, Body hydration and Waist-to-hip ratio were measured on a fasting basis using a body composition analyzer (Inbody720) for participants before and after the intervention.

### 2.4. Blinding

After participants completed the pre-intervention questionnaire and index measures, they were grouped using random numbers, and eligible participants were assigned to the HIIT and AR groups in a 1:1 ratio. Determining the grouping of random numbers was performed by a dedicated study administrator who was not involved in the recruitment and inclusion of participants. Two copies of the random allocation form were placed in an opaque envelope, one for the subject leader and one for the study administrator, and kept in strict confidence. The two copies of the random allocation form will be unsealed in person at the same time when the blind is uncovered at the end of the study.

### 2.5. Data statistics

The results were statistically analyzed using SPSS 19.0 software. Paired samples *t* tests were used to compare within-group prepost comparisons, and one-way analysis of variance (ANOVA) was used to compare between-group differences. Experimental results are expressed as the mean ± standard deviation (Mean ± SD), with *P* < 0.05 indicating a significant difference and *P* < 0.01 indicating a highly significant difference. Furthermore, Cohen's d was calculated as a measure of the effect size.

## 3. Results

**Figure 1:**
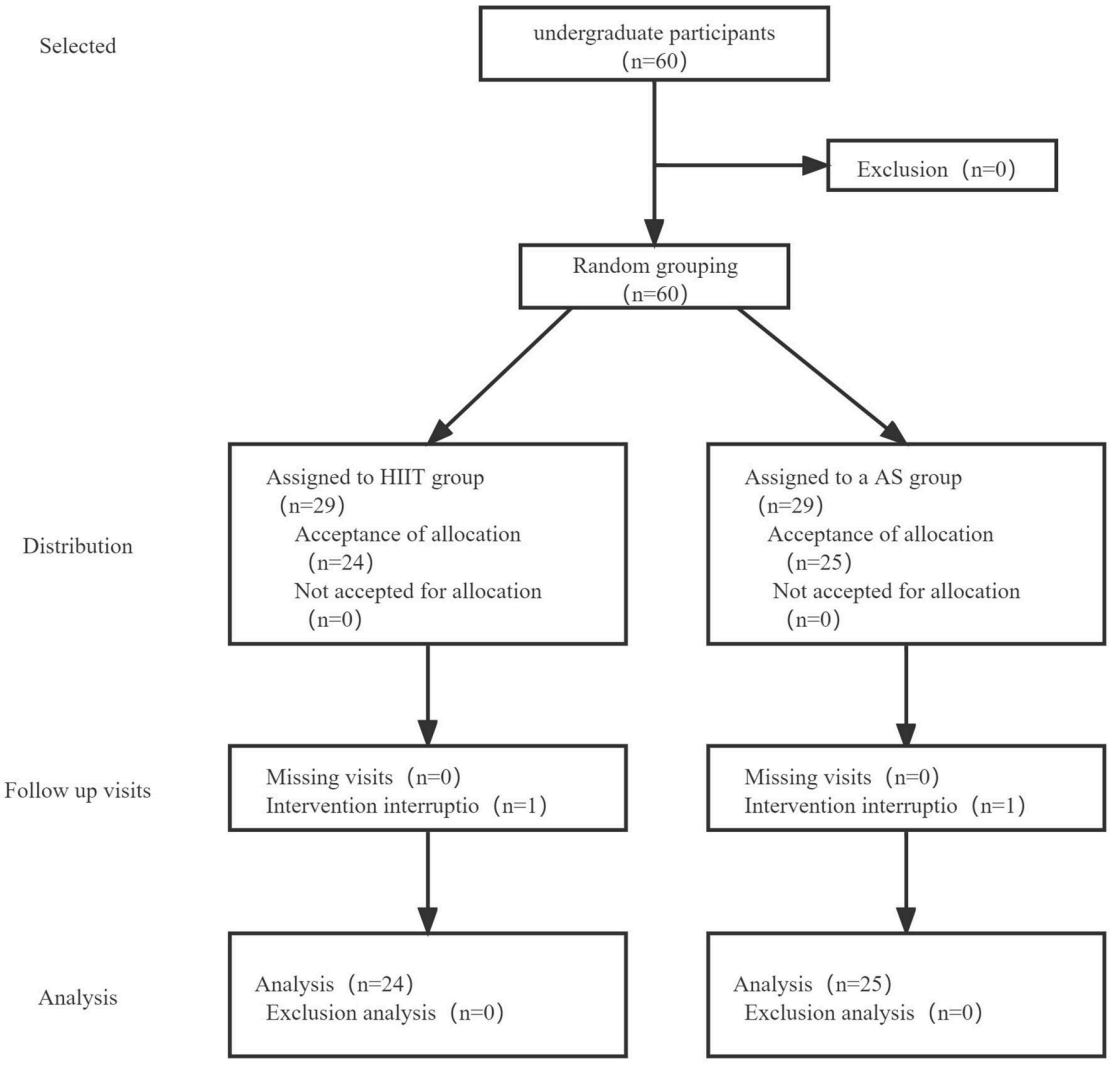
Participants flow chart.

A total of 60 participants participated in the study (Figure 1) and were randomly divided into a HIIT group and a AR group. One student in the HIIT group and one in the AR group discontinued the intervention due to their own problems during the experiment, resulting in a total of 58 students included in the study analysis (*n* = 29 in the HIIT group and *n* = 29 in the AR group).

### 3.1. Basic subject characteristics

As shown in Table 1, between the HIIT and AR groups, the differences in the participants' characteristics were not statistically significant (*P* > 0.05) and met the requirements of the experiment.

**Table 1:** Subject characteristics.

**Features**	**HIIT group (*n* = 29)**	**AR group (*n* = 29)**	***P***
Gender (m/f)	7/15	8/17	0.619
Height (cm)	169.50 ± 7.75	166.41 ± 9.93	0.165
Body weight (kg)	60.27 ± 8.03	58.11 ± 9.15	0.390
BMI (kg/m2)	20.91 ± 1.80	20.88 ± 1.58	0.960
Age (years)	19.91 ± 1.57	19.59 ± 1.53	0.550

Data are expressed as the mean ± standard deviation.

### 3.2. Mental health indicators

The results of the Symptom Self-Rating Scale (SCL-90) and the Pittsburgh Sleep Quality Index (PSQI) tests are shown in Table 2.

**Table 2:** Mental health indicator scores for the HIIT and combined exercise groups.

**Mental health indicators**	**HIIT group**				**AR group**				**Group (*p*)**
	**Pre-test**	**Post-test**	**Intra-group control (p)**	**ES (d-value)**	**Pre-test**	**Post-test**	**Intra-group control (p)**	**ES (d-value)**	
**SCL-90**									
Total score	113.32 ± 16.37	104.41 ± 13.96	0.000^*^	0.59	108.36 ± 12.34	102.08 ± 12.70	0.114	0.50	0.861
Somatization	14.18 ± 2.11	13.00 ± 1.38	0.002^*^	0.66	13.16 ± 1.55	12.76 ± 1.51	0.355	0.26	0.902
Obsessive-compulsive	15.68 ± 3.43	13.05 ± 2.89	0.002^*^	0.83	14.92 ± 3.59	13.56 ± 3.99	0.219	0.36	0.544
Interpersonal sensitivity	12.23 ± 2.78	10.77 ± 2.25	0.010^*^	0.58	11.04 ± 2.33	10.08 ± 1.47	0.130	0.49	0.383
Depression	16.09 ± 2.67	15.09 ± 2.11	0.029^*^	0.42	14.60 ± 2.00	14.44 ± 1.90	0.762	0.08	0.846
Anxiety	11.82 ± 1.82	11.68 ± 1.99	0.731	0.07	10.92 ± 1.91	10.60 ± 1.78	0.610	0.17	0.076
Hostility	6.91 ± 1.51	6.27 ± 0.63	0.045^*^	0.55	6.96 ± 1.10	6.68 ± 1.03	0.410	0.26	0.120
Phobic anxiety	8.32 ± 1.94	8.05 ± 1.76	0.409	0.15	8.00 ± 1.73	7.52 ± 1.01	0.256	0.34	0.270
Paranoiel ideation	7.32 ± 2.19	6.64 ± 1.00	0.074	0.40	7.24 ± 1.48	6.56 ± 0.96	0.077	0.55	0.820
Psychoticism	12.45 ± 2.48	11.09 ± 1.72	0.002^*^	0.64	11.88 ± 1.94	10.80 ± 1.32	0.025^*^	0.65	0.774
Other	8.27 ± 1.20	8.77 ± 1.60	0.094	0.35	8.52 ± 1.56	8.04 ± 1.46	0.270	0.32	0.075
**PSQI**									
Total score	3.64 ± 1.79	4.36 ± 2.74	0.222	0.31	4.56 ± 2.80	4.08 ± 2.38	0.356	0.18	0.309
Subjective sleep quality	0.82 ± 0.59	0.95 ± 0.79	0.378	0.19	0.96 ± 0.74	0.76 ± 0.66	0.203	0.29	0.182
Sleeping time	0.77 ± 0.75	1.00 ± 0.98	0.308	0.26	1.12 ± 1.05	0.96 ± 0.98	0.405	0.16	0.418
Subjective sleep duration	0.18 ± 0.40	0.41 ± 0.59	0.096	0.46	0.48 ± 0.51	0.76 ± 0.78	0.070	0.42	0.361
Sleep efficiency	0.05 ± 0.21	0.23 ± 0.43	0.042^*^	0.53	0.12 ± 0.33	0.00 ± 0.00	0.083	0.42	0.007^*^
Sleep disorders	0.86 ± 0.47	0.73 ± 0.46	0.186	0.28	1.00 ± 0.50	0.80 ± 0.41	0.096	0.44	0.794
Hypnotic drugs	0.09 ± 0.29	0.23 ± 0.53	0.266	0.33	0.04 ± 0.20	0.00 ± 0.00	0.327	0.44	0.047^*^
Daytime dysfunction	0.86 ± 0.77	0.82 ± 0.85	0.847	0.05	0.84 ± 0.80	0.80 ± 0.76	0.832	0.05	0.955

Data are expressed as the mean ± standard deviation. ES, effect size.

* p < 0.05.

SCL-90 test results showed statistically significant differences between the HIIT group after 8 weeks of remote exercise intervention in seven scores: total (*P* = 0.000, ES:d = 0.59), somatization (*P* = 0.002, ES:d = 0.66), obsessive-compulsive (*P* = 0.002, ES:d = 0.83), interpersonal sensitivity (*P* = 0.010, ES:d = 0.58), depression (*P* = 0.029, ES:d = 0.42), hostility (*P* = 0.000, ES:d = 0.59), and psychoticism (*P* = 0.000, ES:d = 0.59). There was a significant decrease in scores for psychopathy in the AR group (*p* = 0.025, ES:d = 0.65), and scores on other test items improved in the posttest, but the difference was not significant (*P* > 0.05, ES:d < 0.5). The results of the covariance between the two groups showed no significant difference between the HIIT and AR groups (*P* > 0.05).

The PSQI test results showed that after 8 weeks of intervention, the scores of all test items in the HIIT group, except for sleep disorders and daytime dysfunction, showed an inverse improvement compared with the pre-intervention results. In the AR group, the scores of all test items except subjective sleep duration decreased to different degrees, and although there was a trend of improvement, it was not statistically significant (*P* > 0.05, ES:d < 0.8).

### 3.3. Physical fitness and body composition indicators

The results of the physical fitness and body composition index tests are shown in Table 3.

**Table 3:** Body composition and fitness test scores for the HIIT and AR groups.

**Body test**	**HIIT group**				**AR group**				**Group (*p*)**
	**Pre-test**	**Post-test**	**Intra-group control (p)**	**ES (d-value)**	**Pre-test**	**Post-test**	**Intra-group control (p)**	**ES (d-value)**	
Maximum oxygen uptake	36.65 ± 8.86	47.21 ± 4.28	0.000^*^	1.52	40.44 ± 4.72	39.32 ± 6.57	0.413	0.20	0.000^*^
Grip strength	29.92 ± 8.01	31.53 ± 9.00	0.031^*^	0.19	30.98 ± 9.87	31.46 ± 9.24	0.526	0.05	0.309
Back muscle strength	77.91 ± 28.07	84.05 ± 28.64	0.093	0.22	74.20 ± 27.81	87.84 ± 26.32	0.000^*^	0.50	0.108
Flexibility	16.03 ± 6.35	17.28 ± 6.04	0.011^*^	0.20	13.24 ± 8.04	14.34 ± 7.53	0.028^*^	0.14	0.494
Body weight	60.27 ± 8.03	58.63 ± 7.92	0.000^*^	0.21	58.48 ± 8.99	57.23 ± 9.26	0.001^*^	0.14	0.371
BMI	20.91 ± 1.80	20.41± 1.59	0.000^*^	0.29	21.01 ± 1.53	20.45 ± 1.63	0.000^*^	0.35	0.697
Body fat percentage	22.39 ± 6.00	20.87± 6.29	0.002^*^	0.25	23.34± 5.90	21.24 ± 6.31	0.000^*^	0.34	0.257
Lean weight	46.96 ± 8.07	46.54± 8.40	0.263	0.05	45.11 ± 9.58	45.38 ± 10.01	0.250	0.03	0.082
Body hydration	34.15 ± 5.92	33.92± 6.21	0.282	0.04	32.86 ± 7.08	33.05 ± 7.38	0.265	0.03	0.077
Waist-to-hip ratio	0.83 ± 0.44	0.81± 0.03	0.001^*^	0.06	0.84 ± 0.03	0.82 ± 0.03	0.000^*^	0.67	0.662

Data are expressed as the mean ± standard deviation. ES, effect size.

* p < 0.05.

The results of the physical fitness index tests showed that in the HIIT group, there were varying degrees of improvement in maximum oxygen uptake, grip strength, back muscle strength and flexibility, with significant differences in all results except for back muscle strength (*P* < 0.05), the d = 1.52 for maximum oxygen uptake, with a large difference. In the AR group, there was no significant improvement in maximal oxygen uptake and grip strength scores, but there were significant differences in back strength and flexibility scores (*P* < 0.05, ES:d < 0.2). The covariance results showed that the HIIT group had a statistically significant difference in maximal oxygen uptake compared to the AR group (*p* = 0.000); the other aspects were not statistically significant.

The results of the body composition indicators tests showed that the HIIT group and the AR group also showed significant differences in body weight, BMI, body fat percentage and waist-to-hip ratio (*P* < 0.01, ES:d < 0.8). In addition, there were no significant differences between the HIIT group and the AR group for any of the outcomes.

## 4. Discussion

The 10 factors in the SCL-90 results reflect each of the 10 areas of psychological symptomatology. A wide range of psychiatric symptomatology is included, ranging from feelings, emotions, thinking, consciousness, and behavior to habits, relationships and eating and sleeping. Both the HIIT and AR groups showed beneficial changes in this scale score. The PSQI results reflect the quality of sleep of the participants, with higher scores indicating poorer sleep quality. The HIIT group and showed an inverse improvement in this scale score and a tendency for the AR group score to decrease. Both the HIIT and AR groups showed varying degrees of improvement in fitness indicators and body composition indicators.

### 4.1. SCL-90 indicators

The results of this study showed that after 8 weeks of remote exercise intervention, participants in both the HIIT and AR groups showed varying degrees of improvement in SCL-90. In the post-test, the mean of the total score was 113.32 ± 16.37 in the HIIT group and 104.41 ± 13.96 in the post-test, while the mean of the AR group was 108.36 ± 12.34 in the pre-test and 102.08 ± 12.70 in the post-test. It can be seen that the HIIT group had a more significant improvement in mental health than the AR group. Several studies have shown that HIIT has a positive effect in improving the mental health of people with mental disorders (Korman et al., 2020; Martland et al., 2020). Scores for somatization, obsessive-compulsive, interpersonal sensitivity, depression, hostility, and psychoticism were also consistently better in the HIIT group than in the AR group. Research suggests that HIIT may improve some aspects of mental health in the general population and may have greater benefits for mental health than other forms of exercise (Martland et al., 2022). Online HIIT exercises can improve the psychological level of university students and effectively reduce stress and depression in university students (Philippot et al., 2022). The HIIT programme has been shown to be effective in reducing stress and depression in university students. With the exception of psychoticism, the total points and all other sub-scores of the AR group were not statistically significant compared to the pre-intervention period, but the mean total and sub-scores were reduced to varying degrees. These results suggest that both HIIT and AR training had a positive impact on improving the mental health of university students, but HIIT may have had a more prominent effect on improving the mental health of university students.

### 4.2. PSQI indicators

Vgontzas et al. (2012) showed that sleep is one of the most important factors affecting mental health and that sleep problems can lead to various psychological problems; Passos et al. (2023) found that exercise was effective in improving sleep quality and reducing depressive symptoms. Previous studies have also shown that (Ezati et al., 2020) regular physical activity can improve sleep duration and sleep efficiency, which in turn can improve sleep quality. Sleep quality is a composite of the physical and mental health of university students (Wang et al., 2016). After 8 weeks of remote exercise intervention, the HIIT group showed varying degrees of improvement in sleep quality indicators, except for sleep disorders and daytime dysfunction. Previous studies have shown that HIIT is effective in improving sleep quality and efficiency in adults (Leizi et al., 2021). However, the results of this study showed that HIIT had a negative impact on sleep quality in the participants, possibly due to the following reasons: (1) The HIIT group exercised for too long, the total amount of exercise was too large, and the design of the exercise prescription was flawed. (2) Due to the students' busy schedule during the day and the specific nature of the remote intervention, the majority of student-directed interventions were concentrated in the evening, and too much exercise led to sympathetic and parasympathetic excitation and thus affected their sleep. Although the total PSQI score and other sub-scores in the AR group were not statistically significant compared with the pre-intervention period, the mean values of the total and sub-scores were reduced to different degrees. This suggests that combined exercise can contribute to the improvement of sleep quality in university students. The principles of sport psychology suggest that moderate- to low-intensity exercise for 20–60 min 3–5 times per week can significantly improve mental health. The amount of exercise in the AR group was largely in line with the optimal exercise prescription, and therefore, the final PSQI score showed a beneficial change. Previous studies have shown that regular aerobic exercise can improve psychomotor performance and sleep quality in professional athletes (Taheri and Valayi, 2018). Therefore, combined exercise may have a better sleep-promoting effect than HIIT.

### 4.3. Physical fitness indicators

Maximum oxygen uptake is considered to be one of the best indicators of cardiorespiratory fitness (Poole and Jones, 2017). After 8 weeks of tele-exercise intervention, there was a significant improvement in maximal oxygen uptake in the HIIT group (*P* = 0.000, ES:d = 1.52), and the mean value of maximal oxygen uptake in the HIIT group improved from 36.65 ± 8.862 on the pre-test to 47.21 ± 4.278 on the post-test, with a more significant increase in the score. The mean value of maximal oxygen uptake in the AR group decreased from 40.44 ± 4.719 in the pre-test to 39.32 ± 6.568 in the post-test, showing that the HIIT group had better results than the AR group after the intervention. One of the earliest studies (Baquet et al., 2001) suggested that HIIT could improve aerobic exercise capacity in children and may be more effective in improving their fitness. Dias et al. (2018) 12-week intervention with obese children found that HIIT interventions were effective in improving cardiorespiratory fitness compared to MICT and nutritional interventions and that HIIT could be an effective health strategy to improve physical fitness in adolescents. Costigan et al. (2015) found that high-intensity interval training improved the cardiorespiratory fitness of university students. Previous studies have shown that 30 min of HIIT training per week is effective in improving maximal oxygen uptake (Reljic et al., 2018). For the muscle strength index, the results of the between-group covariance showed no significant difference in change between the HIIT and AR groups. However, there was a significant improvement in grip strength in the HIIT group (*P* = 0.031, ES:d = 0.19) and in back muscle strength in the AR group (*P* = 0.000, ES:d = 0.50). Flexibility was significantly improved in both the HIIT and AR groups (*P* = 0.011, ES:d = 0.20; *P* = 0.028, ES:d = 0.14). Taheri et al. (2021) found significant improvements in physical flexibility using aquatic therapy and jogging exercises. The present results suggest that HIIT and combined exercise are beneficial for the improvement of flexibility in college students.

### 4.4. Body composition indicators

Body composition refers to the content and proportion of each body component. The results showed that after 8 weeks of remote exercise intervention, participants in both the HIIT and AR groups had varying degrees of change in body composition. Some studies have shown that 4 weeks of HIIT can quickly and effectively improve body composition in overweight female university students (Guo et al., 2022). However, some studies have also shown that HIIT has no significant effect on body composition indicators (Irandoust et al., 2022). The results of the between-group covariates showed no significant differences between the HIIT and AR groups after 8 weeks of the remote intervention. However, both the HIIT and AR groups showed significant improvements in several areas of body weight, BMI, body fat percentage, and waist-to-hip ratio. This suggests that physical activity has significant benefits in terms of reducing fat content. Foreign studies (Florie et al., 2018) have confirmed that HIIT is effective in reducing adiposity and body fat percentage. Akhoundnia et al. (2019) showed that performing wrestling-based HIIT for 8 weeks significantly improved body composition, particularly body fat percentage and body weight. García-Hermoso et al. (2018) showed that aerobic exercise combined with resistance exercise can improve body composition and metabolic status and promote adolescent health in obese children. These studies are consistent with the results of this study. In addition, the combined exercise group showed a slight improvement in lean weight, and Villareal et al. (2017) confirmed that aerobic exercise combined with resistance training increased lean body mass more significantly.

## 5. Limitations

The sample size of this study is small, and the intervention schedule is affected by the students' daily study and life, which may affect the experimental results, and a more reasonable intervention plan as well as a larger sample size is needed for the study.

## 6. Conclusion

In summary, the results of this study suggest that HIIT shows a more beneficial effect on the mental health of university students. However, further studies with larger sample sizes and longer intervention durations are needed to confirm its role in the mental health of university students. Remote coaching HIIT and combined training have shown some improvement in fitness levels and body composition in university students, HIIT is better at improving aerobic endurance, and remote coaching HIIT based on mental health may be better than combined exercise, but more rigorous controlled trials are still needed to confirm this.

## Data availability statement

The original contributions presented in the study are included in the article/supplementary material, further inquiries can be directed to the corresponding author.

## Ethics statement

The studies involving human participants were reviewed and approved by College of Physical Education, Shandong Normal University, Jinan, China. The patients/participants provided their written informed consent to participate in this study. Written informed consent was obtained from the individual(s) for the publication of any potentially identifiable images or data included in this article.

## Author contributions

BL planned the structure of the manuscript. YW carried out the study, collected the important background information, and drafted the manuscript. NJ, YZ, and LFu assisted in data acquisition, data analysis, and statistical analysis. LFa reviewed and edited the final manuscript. All authors have read and approved the final manuscript.
